# Higher stress response and altered quality of life in schizophrenia patients with low membrane levels of docosahexaenoic acid

**DOI:** 10.3389/fpsyt.2023.1089724

**Published:** 2023-02-03

**Authors:** Vladimir Adrien, Nicolas Bosc, Hugo Fumat, Cédric Tessier, Florian Ferreri, Stéphane Mouchabac, David Tareste, Philippe Nuss

**Affiliations:** ^1^AP-HP, Sorbonne Université, Department of Psychiatry, Hôpital Saint-Antoine, Paris, France; ^2^Infrastructure for Clinical Research in Neurosciences (iCRIN), Paris Brain Institute, Sorbonne Université, INSERM, CNRS, Paris, France; ^3^Université Paris Cité, INSERM UMR-S 1266, Institut de Psychiatrie et Neurosciences de Paris, Paris, France; ^4^Centre de Recherche Saint-Antoine, INSERM UMR S938, Sorbonne Université, Paris, France

**Keywords:** schizophrenia, membrane fatty acids, biomarkers, quality of life, stabilized patients

## Abstract

Schizophrenia is a severe, chronic, and heterogeneous mental disorder that affects approximately 1% of the world population. Ongoing research aims at clustering schizophrenia heterogeneity into various “biotypes” to identify subgroups of individuals displaying homogeneous symptoms, etiopathogenesis, prognosis, and treatment response. The present study is in line with this approach and focuses on a biotype partly characterized by a specific membrane lipid composition. We have examined clinical and biological data of patients with stabilized schizophrenia, including the fatty acid content of their erythrocyte membranes, in particular the omega-3 docosahexaenoic acid (DHA). Two groups of patients of similar size were identified: the DHA− group (*N* = 19) with a lower proportion of membrane DHA as compared to the norm in the general population, and the DHAn group (*N* = 18) with a normal proportion of DHA. Compared to DHAn, DHA− patients had a higher number of hospitalizations and a lower quality of life in terms of perceived health and physical health. They also exhibited significant higher interleukin-6 and cortisol blood levels. These results emphasize the importance of measuring membrane lipid and immunoinflammatory biomarkers in stabilized patients to identify a specific subgroup and optimize non-pharmacological interventions. It could also guide future research aimed at proposing specific pharmacological treatments.

## 1. Introduction

Schizophrenia is a serious and chronic psychiatric disease with a prevalence of 0.28–0.66% and an incidence of 10–22 new cases per year for 100,000 people (1–3). It is a heterogeneous disease in terms of symptomatology, outcome, treatment response, and etiopathogenesis, involving neurodevelopmental anomalies in a context of polygenic vulnerability and gene-environment interaction (4–7). Numerous phenomena, either acute or chronic, are at play and vary during the life-long course of the disorder, including both brain and whole-body processes. In addition to drug prescriptions to help regulate these processes, non-pharmacological interventions, such as rehabilitation and patient empowerment programs, are proposed to optimize the overall treatment trajectory. Most treatment guidelines for clinicians are based on neuropharmacological studies conducted during the short period corresponding to the acute phase of the disorder, with comparatively fewer data on the best drug adjustments in terms of dosage and combinations during the stabilization phase.

The rationale for the pharmacological treatment of schizophrenia is based on the assumption of a dysfunctional dopamine brain signaling that comes along with structural defects in striato-cortico-thalamic pathways (8–10). Current research shows that many regulation systems, central or peripheral, are altered in schizophrenia (11, 12). These perturbations, which are sometimes moderate, or even present in healthy subjects, involve minor physical anomalies (13, 14), minor neurological signs (15, 16), oxidative stress (17–19), inflammation and immunity (20–23), lipid metabolism (24, 25), and alteration of the hormonal system (26), microbiota (27), or microglia (28, 29). Many studies have also shown the existence of membrane fatty acid, phospholipid, and glycolipid abnormalities, as well as lipidic signaling defects, associated with symptom severity in schizophrenia (30–35).

In this manuscript, we focused on two polyunsaturated fatty acid (PUFA) families with distinct properties. The omega-3 (ω3) family is mainly composed of alpha-linoleic acid (ALA), eicosapentaenoic acid (EPA), and docosahexaenoic acid (DHA) that have anti-inflammatory properties. The omega-6 (ω6) family includes linoleic acid (LA), arachidonic acid (AA), and docosapentaenoic acid (DPA) that have pro-inflammatory properties (36). Therefore, the ω6/ω3 ratio in dietary intake is critical.

Several studies on human subjects focused on the PUFA content of the red blood cell (RBC) membranes (30–33, 37), which has been shown to correlate with the PUFA content in the brain (38–42). These studies found a decrease of DHA and AA in the red blood cell (RBC) membranes from a subgroup of patients with schizophrenia, and observed that this decrease was associated with more severe symptoms. A decrease in DHA and AA, together with a lower level of EPA, was also observed in patients with an Ultra-High Risk (UHR) of psychotic transition, defined by the Comprehensive Assessment of At-Risk Mental States (CAARMS) (43). A study examining individuals with first schizophrenic episodes showed that ω3, but not ω6, rates were decreased compared to healthy controls (44). On the other hand, this study did not observe any differences in ω3 or ω6 rates between healthy individuals and subjects with chronic schizophrenia who did not receive any pharmacological treatment. This study also showed that treatment with atypical antipsychotics (AP) could partly correct the ω3 abnormality in first episode patients. Overall, this suggests that a deficiency in ω3 could be present at the beginning of the disease in some schizophrenia patients but would not persist over time.

Clinical trials have been made with ω3 supplementation in acute or stabilized patients with controversial results. Some studies showed some clinical improvement (45) while others could not evidence any efficacy of a supplementation with ω3 on symptoms or functionality (46). A monocentric study, however, found that dietary supplementation of ω3 was able to reduce the risk of psychotic transition in UHR patients (47). In this study, psychotic transition rates stayed weak, as well as compliance to diet, making it difficult to draw any conclusion. Many studies however propose to combine ω3 supplementation with AP treatment in order to improve the therapeutic effect of AP (48–50).

For stabilized patients, it is well-acknowledged that medication maintenance, cognitive therapies, and rehabilitation programs are critical for symptom reduction, and improvement of functionality and quality of life. Strikingly, whereas for the most part of their life, patients are not in an acute phase of the disease, there is a lack of biological data to guide pharmacological and non-pharmacological interventions during the stabilization phase (51). It is most probable that the biological signature of treated stabilized patients differs from that existing during the acute exacerbation of the disease. It could thus be interesting to pay attention not only to the clinical and functional response of stabilized patients, but also to their biomarkers. This could allow the clinician to biologically define a stabilization profile and identify the parameters that should be addressed to optimize the care.

The purpose of the present paper is to examine to what extent blood biomarkers coupled with clinical and cognitive data can contribute to disentangle disease heterogeneity and improve treatment in clinically stabilized schizophrenic patients. We retrospectively reviewed data from well-stabilized patients followed during several months in the Saint-Antoine psychiatric day hospital. We notably examined whether the PUFA level in the RBC membranes of these patients correlated with symptom severity, quality of life and other biological markers.

## 2. Materials and methods

### 2.1. Subjects

Medical records of 37 patients with criteria of schizophrenia (14 females, mean age: 44 years) have been retrieved. This was the exhaustive cohort of patients having been admitted to the psychiatric department of the Saint-Antoine day hospital in Paris, France in the 2 years prior to the initiation of the study. The indications for day hospitalization were symptoms stabilization, and implementation of rehabilitation and occupational therapy programs. At the time of assessment, all patients were living autonomously and could manage their medication by themselves. Most came part-time. They were receiving psychopharmacological treatment and were stabilized (i.e., at the time of assessment, all were admitted to the day hospital and had no recent significant change in their medication).

Inclusion criteria were being 18 years old or older, having criteria for schizophrenia as assessed by the Diagnostic and Statistical Manual of Mental Disorders, Fifth Edition (DSM-5), and having signed a written informed consent for the use of medical data in their electronic health record for research [AP-HP Health Data Warehouse (EDS), which has been authorized since December 22, 2021 by the authorization n° 20211222104100].

Exclusion criteria were central neurological disease, recent major changes in their medication (i.e., a change of more than 20% of the current medication dose or the addition of another treatment) or modification in their diet or physical activity in the 3 months preceding the clinical and biological assessment. Significant medication or habit changes were considered as a proxy of disease destabilization or likely to have an impact on biological markers such as metabolic-energetic markers, especially regarding lipid metabolism. Patients were under a wide variety of psychopharmacological medications depending on their treatment history. Previous studies have shown that antipsychotics have variable effects on FA levels. Some studies found no effect (30, 37, 52) while others observed little effect (31, 53) of AP treatment on FA levels, which seemed to depend on the type of AP (typical vs. atypical) used and/or the phase (acute vs. chronic) of the disease under investigation (54, 55). In our case, because the sample size was too low, we could not make any correlation between PUFA levels and type of treatment or number of episodes.

### 2.2. Evaluation of medical history

During their stay in the day hospital, all patients underwent extensive biological and psychological tests, similar to those performed during a full-time hospitalization in the same department. Retrieved data included sociodemographic information (age, sex, weight, height, abdominal perimeter, level of education, etc.). Clinical evaluation included criteria for schizophrenia as well as psychiatric comorbidities, suicide attempts history and number, family history of schizophrenia, schizoaffective disorder, or substance use disorders. A specific focus was made on diet issues, particularly supplementation in PUFAs. The course of the schizophrenia was also examined including age at first symptoms, first medical consultation, first hospitalization, and first AP treatment, as well as the number and average length of hospitalizations, and the treatment history.

### 2.3. Symptoms, cognition, and lifestyle assessment

Data were collected using both self- and clinician-rated questionnaires. Self-questionnaires were all in their French version and evaluated: (1) the type of diet based on a questionnaire used by the French national diet enquiry (56), (2) the quality of life with the Short Form (SF-36) Health Survey (57, 58) and the Duke Health Profile (59), (3) the physical activity, quantified by two scales, the Ricci and Gagnon (60) and the Modifiable Activity Questionnaire (MAQ) (61–64), and (4) the functional impairment with the Sheehan Disability Scale (SDS) (65). All scales were validated in French (58, 59, 66) except the French national diet enquiry, the Ricci and Gagnon scale and the SDS.

Clinician-evaluated questionnaires were also in their French version and included: (1) the Clinical Global Impression–Severity (CGI-S) scale (67), (2) the Global Assessment Functioning (GAF) scale (68), (3) the Positive and Negative Syndrome Scale (PANSS) (69, 70), (4) the Calgary Depression Scale for Schizophrenia (CDSS) (71, 72), (5) the Montreal Cognitive Assessment (MoCA), version 7.1 (73, 74), and (6) the Modified Wisconsin Card Sorting Test (MCST) (75–80). When the patient had already been tested by the MoCA, one of the two re-test versions of the MoCA was used. The MCST was rated at the end of the systematic evaluation of patients because it can bring moderate anxiety that could disturb the rating of other scales. The PANSS, CDSS and MoCA questionnaires were validated in French (72, 81, 82).

### 2.4. Biological data

Retrieved biological and treatment data included: AP or mood stabilizers blood dosage, standard biology [blood count, serum electrolytes, creatine phosphokinase (CPK)], metabolic syndrome markers (LDL-cholesterol, HDL-cholesterol, triglycerides, fasting blood sugar, glycated hemoglobin), hormonal assays [Thyroid-Stimulating Hormone (TSH), prolactin], inflammation markers [Ultra-Sensitive C-Reactive Protein (US-CRP), Interleukin-1β (IL-1β), Interleukin-6 (IL-6), TNFα], energetic markers (amino acids among which homocysteine and methionine, B6, B9, and B12 vitamins), and finally lipidomic analysis [complete steroid profile, serum bile acids, RBC membrane FA].

### 2.5. Gas chromatography coupled with mass spectrometry (GC-MS) analysis of RBC membrane FA composition

After RBC lipid extraction following previously established protocols (83), the RBC membrane FA composition was obtained by GC-MS in the positive chemical ionization mode with ammonia as the reagent gas (GC 6890—MS 5975; Agilent) (84). The calibration of the response factors for FAs was made with a weighed mixture (FAME Mix Supelco47^®^, Sigma-Aldrich Chemie) (85). Profiles are expressed as mol%.

### 2.6. Statistics

Descriptive statistics include usual parameters: mean ± standard deviation (S.D.) or number of subjects and percentages. Data were expressed as mean ± SD, number and percentages, and analyzed using Epi Info™ software (7.2.1.0 version) from the Centers for Disease Control and Prevention, Atlanta, USA.^1^ Patients’ characteristics correlations with RBC DHA class were investigated using Spearman’s correlation tests. χ2 tests were performed for nominal variables and independent samples *T*-test for continuous variables. All the tests were two-sided with a statistical significance level set at *p* = 0.05. With a 5% alpha risk, DHAn and DHA− samples of sizes 18 and 19, respectively, allow for the identification of effect sizes of 0.82, 0.92, and 1.06 with 70, 80, and 90% powers, respectively. This means that considering that an 80% power is satisfactory, the sample size may be considered as sufficient when the hypothesized difference between groups is at least 92% of the standard deviation for this variable.

## 3. Results

Sociodemographic and clinical characteristics of patients are reported in Table 1. Among the 37 patients, 23 (62%) were men. The mean age of the cohort was 44 years. The first psychotic clinical manifestations appeared at a mean age of 20 years, while the mean age at first hospitalization was 24 years. The mean total number of hospitalizations was 7.7 with an average length of hospital stay of 42 days. A total of 13 (35%) patients had a history of suicide attempts. In the cohort, 6 (16%) patients had a first degree family history of schizophrenic or schizoaffective disorders, and 11 (30%) had a second degree or above. In addition, 21 (57%) patients were active smokers, and 5 (14%) had criteria for alcohol disorder.

**TABLE 1:** Sociodemographic and clinical characteristics of the patient population, comparing the two groups DHAn and DHA−.

	Total (*n* = 37)	DHAn (*n* = 18)	DHA− (*n* = 19)	RR [95% CI]	*P*-value
Male gender	23 (62%)	11 (61%)	12 (63%)	1.05 [0.53–2.05]	0.58
Age (years)	44 (10)	40 (10)	47 (9.1)		0.026
BMI (kg/m^2^)	28.6 (5.7)	28.7 (5.6)	28.5 (5.9)		0.94
Education	13.2 (3.3)	14.4 (3.1)	12.0 (3.1)		0.030
Schizo-affective disorder	21 (57%)	10 (56%)	11 (58%)	1.05 [0.54–2.04]	0.57
Suicide attempt	13 (35%)	6 (33%)	7 (37%)	1.08 [0.53–2.20]	0.55
1st degree background	6 (16%)	4 (22%)	2 (11%)	0.68 [0.34–1.35]	0.30
>1st degree background	11 (30%)	5 (28%)	6 (32%)	1.1 [0.52–2.34]	0.54
Active tobacco use	21 (57%)	8 (44%)	13 (68%)		0.13
Daily alcohol intake (g/day)	5 (14%)	1 (5.6%)	4 (21%)	2.65 [0.45–15.80]	0.19
Daily THC intake (reefer/day)	5 (14%)	2 (11%)	3 (16%)	1.25 [0.40–3.86]	0.53
Age 1st symptoms (years)	20 (7.6)	21 (5.5)	20 (9.4)		0.79
Age 1st consultation (years)	23 (8.2)	22 (5.8)	24 (10)		0.60
Age 1st hospitalization (years)	24 (8.1)	23 (6.0)	25 (9.8)		0.56
Number of hospitalizations	7.7 (10)	3.4 (3.7)	12 (13)		0.02
Average length of stay (days)	42 (49)	35 (36)	48 (60)		0.44

RR, relative risk; 95% CI, confidence interval; THC, tetrahydrocannabinol; BMI, body mass index. If the result is a number of patients, the percentage is between brackets. If the result is an average value, the standard deviation is between brackets. There are no statistical differences between the two groups, except for the age, the level of education and the number of hospitalizations.

The fatty acid composition of RBC membranes from patients was determined by Mass Spectrometry (Table 2). The rate of the ω6 AA was increased in patients, with a mean value of 16.2%, as compared to the rate of the general population which is between 12.7 and 14.7% (86). Only 4 patients presented a normal AA rate. The rate of the ω3 DHA was slightly lowered, with a mean value of 5.6%, whereas the normal rate is between 5.7 and 7.8%. ALA rate was also lowered, with an average of 0.11%, for a normal rate between 0.2 and 0.3%.

**TABLE 2:** Comparison of the RBC membranes fatty acid (FA) composition, hormonal markers and inflammation markers from patients of the two groups.

	Total (*n* = 37)	Norm	DHAn (*n* = 18)	DHA− (*n* = 19)	*P*-value
**RBC membranes FA**					
EPA (%)	0.87 (0.34)	0.7–1.6	1.00 (0.42)	0.75 (0.2)	0.02
DHA (%)	5.6 (1.1)	5.7–7.8	6.5 (0.7)	4.8 (0.7)	<0.001
ALA (%)	0.11 (0.03)	0.2–0.3	0.11 (0.03)	0.11 (0.04)	0.75
AA (%)	16.2 (1.3)	12.7–14.7	16.0 (1.1)	16.4 (1.4)	0.31
LA (%)	9.8 (1.2)	8.9–10.9	9.7 (0.9)	10 (1.4)	0.40
Sum ω3 (%)	9.0 (1.6)		10.2 (1.0)	7.8 (1.0)	<0.001
Sum ω6 (%)	29 (1.6)		29 (1.5)	30 (1.6)	0.044
Ratio ω6/ω3	3.3 (0.6)		2.9 (0.4)	3.8 (0.5)	<0.001
**Hormonal markers**					
Prolactine for all patients (μg/L)	20.9 (19.2)	<29 <20 after menopause	23.5 (23.1)	18.5 (14.7)	0.44
Testosterone among men (nmol/L)	14.0 (6.9)	9–38	13.8 (6.9)	14.2 (7.2)	0.90
DHEA among women (nmol/L)	14.1 (5.5)	8–35	15.4 (7.3)	12.8 (3.0)	0.40
DHEA among men (nmol/L)	20.0 (9.6)	8–35	16.9 (8.1)	22.7 (10.4)	0.15
Cortisol for all patients (nmol/L)	336.2 (126.5)		293.3 (79.6)	376.7 (149.9)	0.043
Progesterone among women (nmol/L)	5.12 (7.80)		4.22 (8.47)	6.61 (8.02)	0.71
**Inflammation markers**					
US-CRP (mg/L)	4.0 (4.9)	<5	3.7 (4.7)	4.4 (5.3)	0.71
IL-1β (pg/mL)	13.4 (28.2)	<15	11.3 (30.5)	15.5 (26.5)	0.65
IL-6 (pg/mL)	6.4 (16.4)	<8.5	0.072 (0.306)	12.4 (21.4)	0.020
TNFα (pg/mL)	23.2 (18.8)	<20	21.9 (8.2)	24.5 (25.3)	0.68

Standard deviations are between brackets. EPA (precursor of DHA), DHA and ω3 FA as a whole are in smaller proportion in the DHA− group, whereas the proportion of ω6 FA is increased in this group, as well as the ω6/ω3 ratio. Prolactin and cortisol were analyzed for both genders at the same time because no differences were found between the two genders. There is an increase of cortisol (*p* = 0.043) in the DHA− group. We also measured a much higher level of IL-6 in the DHA− group (*p* = 0.020). Sum ω3, sum of ALA, EPA, DHA, and docosapentaenoic (C22:5 n-3) acid; Sum ω6, sum of LA, AA, docosatetraenoic, and docosapentaenoic (C22:5 n-6) acids; DHEA, dehydroepiandrosterone.

Dosages of hormonal and inflammation markers among patients are reported in Table 2. Only TNFα was increased for all patients, with an average rate of 23.2 pg/mL, while the normal rate is under 20 pg/mL.

Results on the perceived quality of life are given in Table 3, while Table 4 reports psychopathology and cognitive scores. As expected in this stabilized population, the GAF score was on average at 51, corresponding to moderate symptoms or moderate difficulties in socio-professional functioning. Similarly, the average PANSS was rated at 81.0, the Calgary at 16.0 and the MoCA at 24.9, corresponding to mild or moderate psychotic, depressive and cognitive symptoms.

**TABLE 3:** Results on the quality of life comparing the two groups.

	Total (*n* = 37)	DHAn (*n* = 18)	DHA− (*n* = 19)	*P*-value
GAF (/100)	51 (11)	53 (8)	48 (13)	0.32
**SF-36**				
Perceived health (/100)	51 (21)	62 (19)	42 (20)	0.0037
Evolution perceived health (/100)	28 (29)	21 (27)	34 (30)	0.17
Physical activity (/100)	79 (18)	89 (9)	69 (20)	<0.001
Physical limitation (/100)	55 (35)	60 (32)	50 (37)	0.40
Physical pain (/100)	76 (22)	84 (19)	69 (22)	0.037
Mental health (/100)	55 (16)	57 (15)	53 (16)	0.44
Mental limitation (/100)	65 (38)	56 (38)	74 (38)	0.15
Social relations (/100)	68 (24)	75 (23)	61 (23)	0.066
Vitality (/100)	47 (16)	50 (15)	44 (16)	0.28
**Duke**				
General health (/100)	54 (23)	62 (20)	47 (24)	0.053
Physical health (/100)	65 (21)	72 (16)	58 (23)	0.036
Mental health (/100)	51 (26)	52 (25)	49 (27)	0.80
Social relations (/100)	55 (21)	59 (17)	52 (25)	0.31
Perceived health (/100)	47 (41)	61 (40)	34 (37)	0.043
Incapacity (/100)	5.4 (16)	5.6 (16)	5.3 (16)	0.96
Anxiety (/100)	47 (24)	44 (22)	49 (26)	0.52
Self esteem (/100)	54 (23)	60 (24)	47 (21)	0.10
Depression (/100)	45 (25)	44 (24)	46 (26)	0.77
Pain (/100)	39 (38)	28 (26)	50 (44)	0.071
**SDS**				
Work (/10)	5.2 (3.8)	4.5 (3.3)	6.0 (4.2)	0.27
Social (/10)	5.1 (3.7)	4.9 (3.7)	5.3 (3.7)	0.80
Family (/10)	4.1 (3.2)	3.7 (3.2)	4.6 (3.3)	0.40

Standard deviations are between brackets. On the SF-36 scale, the DHA− group has a lower quality of life in terms of perceived health (*p* = 0.0037), physical activity (*p* < 0.001), and physical pain (*p* = 0.037). On the Duke scale, the DHA− group has lower physical health (*p* = 0.036) and perceived health (*p* = 0.043). There are no statistical differences between the two groups on the Global Assessment of Functioning (GAF) or the Sheehan Disability Scale (SDS).

**TABLE 4:** Results on the neuropsychiatric symptoms comparing the two groups.

	Total	DHAn (*n* = 18)	DHA− (*n* = 19)	*P*-value
CGI-S (0–7)	4.6 (0.7)	4.5 (0.5)	4.6 (0.9)	0.58
**Psychotic symptoms (PANSS)**				
Positive (7–49)	18.0 (5.4)	18.5 (5.4)	17.5 (5.5)	0.59
Negative (7–49)	21.6 (4.9)	21.9 (5.5)	21.4 (4.4)	0.73
Psychopathology (16–112)	41.4 (8.0)	41.8 (7.9)	40.9 (8.4)	0.74
Total PANSS (30–210)	81.0 (13.7)	82.3 (13.3)	79.8 (14.4)	0.60
**Depressive symptoms (CDSS)**				
Calgary (0–27)	16.0 (4.6)	14.9 (3.8)	17.1 (5.1)	0.15
**Cognitive symptoms (MoCA)**				
Visuospatial/executive (0–5)	3.7 (1.2)	3.8 (0.9)	3.7 (1.5)	0.82
Naming (0–3)	3.0 (0.2)	2.9 (0.2)	3.0 (0.0)	0.31
Attention (0–6)	4.9 (1.4)	5.3 (1.2)	4.6 (1.5)	0.14
Language (0–3)	2.5 (0.6)	2.6 (0.5)	2.4 (0.6)	0.19
Abstraction (0–2)	1.6 (0.6)	1.5 (0.8)	1.7 (0.5)	0.39
Delayed recall (0–5)	4.0 (4.9)	3.6 (1.3)	4.5 (6.9)	0.58
Orientation (0–6)	5.8 (0.4)	5.8 (0.5)	5.8 (0.4)	0.78
Total MOCA (0–30)	24.9 (3.8)	25.7 (3.7)	24.2 (3.8)	0.21
**Cognitive symptoms (MCST)**				
Correct categories (0–6)	4.9 (1.3)	4.9 (1.2)	4.8 (1.4)	0.88
Cards for 6 categories (n)	42.8 (3.5)	41.8 (3.6)	43.9 (3.4)	0.24
Mistakes (n)	10.1 (5.8)	10.1 (6.7)	10.0 (4.9)	0.96
Perseverations (n)	3.6 (3.3)	3.7 (3.8)	3.6 (2.7)	0.94
PAC (n)	2.3 (2.5)	2.3 (2.2)	2.4 (2.8)	0.98
% perseverations	33 (25)	32 (28)	34 (22)	0.83

PAC, premature abandonment of the criteria. Psychotic, depressive, or cognitive symptoms do not differ between the two groups.

**FIGURE 1:**
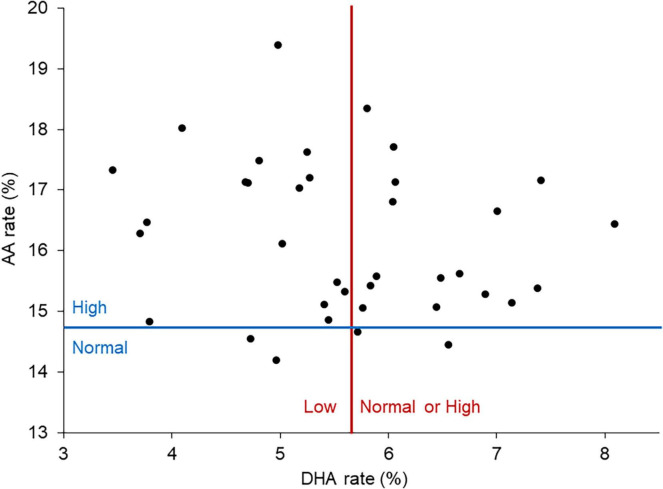
DHA and AA rates among patients. The red line corresponds to the threshold between low and normal or high DHA rates. The blue line corresponds to the threshold between normal and high AA rates. Almost all patients had an elevated AA level. Patients were divided in two groups of comparable size based on their DHA level: DHAn corresponding to a normal or high DHA rate and DHA– corresponding to a low DHA rate.

The evidence of an increased AA mean rate along with a decreased DHA mean rate, prompted us to examine the AA and DHA distribution within the studied cohort. AA rates were high in almost all patients (*N* = 33), whereas DHA rates were low in only half of the patients (*N* = 19). We thus chose to split the cohort into 2 subgroups of patients according to their DHA rate. The group named DHAn (*N* = 18) was constituted of patients whose RBC membrane DHA content was in the range of the norm (*N* = 17) or above (*N* = 1), and the group named DHA− (*N* = 19) was composed of patients with a low DHA rate (Figure 1).

Patients in the DHA− group were older (47 vs. 40 years, *p* = 0.026), and had a lower level of education (12.0 vs. 14.4, *p* = 0.03) (Table 1). They had a higher number of hospitalizations (12 vs. 3.4, *p* = 0.02) but the length of full-time hospital stay was identical.

There was no statistically significant difference between the two groups in terms of duration of untreated psychosis (DUP), considered either as the duration between the first symptoms and the first consultation (3.89 vs. 1.85 years, *p* = 0.25), or between the first symptoms and the first hospitalization (4.67 vs. 2.32 years, *p* = 0.20).

No significant differences were measured between DHA− and DHAn groups in terms of sex ratio, body mass index, proportion of schizoaffective disorder diagnosis, suicidal attempts, or first (or more) degree family history of schizophrenia or schizoaffective disorder. Likewise, no significant differences were found concerning comorbid substance uses, or age of illness onset (first symptoms, consultation, or hospitalization). There were also no statistical differences between the two groups in terms of diet habits and physical activity (Supplementary Table 1).

Consistent with the membrane DHA ratio decrease, we found a significant decrease of the ω3 EPA (0.75 vs. 1.00, *p* = 0.02) and of the total ω3 content (7.8 vs. 10.2, *p* < 0.001) in patients from the DHA− group, whereas the total ω6 content was slightly increased (30 vs. 29, *p* = 0.044) (Table 2). The ω6/ω3 ratio was also different between the two groups with a higher ratio in the DHA− group (3.8 vs. 2.9, *p* < 0.001).

The two groups did not differ significantly in metabolic-energetic markers values (Supplementary Table 2), such as LDL-cholesterol, HDL-cholesterol, triglycerides, fasting blood sugar, glycated hemoglobin, B6, B9, B12 vitamins, amino acids, and bile acids.

**FIGURE 2:**
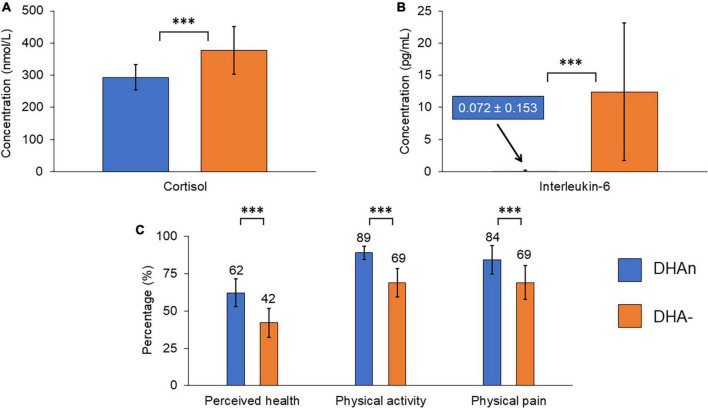
Summary graph of the main differences observed between the two groups of patients: **(A)** Cortisol level, **(B)** interleukin-6 level, and **(C)** quality of life as measured by the SF-36 scale. The cortisol and interleukin-6 levels are increased in the DHA– group. Quality of life dimensions represented here are decreased in the DHA– group. Error bars are standard deviations. ***Means *p* < 0.05 by χ^2^ test for nominal variables and *T*-test for continuous variables.

Interestingly, patients in the DHA− group had a higher rate of cortisol (376.7 vs. 293.3 nM, *p* = 0.043, see also Figure 2A). No gender differences were found in prolactin and cortisol (Table 2).

Finally, concerning inflammation markers (Table 2 and Figure 2B), IL-6 rates were dramatically increased in the DHA− group (12.4 vs. 0.072 pg/mL, *p* = 0.020), whereas no statistical difference was observed for TNFα, IL-1β, or US-CRP between the two DHA groups.

Quality of life measurements assessed with the SF-36 scale (Table 3 and Figure 2C) showed a lower perceived health (42 vs. 62%, *p* = 0.0037), lower physical activity (69 vs. 89%, *p* < 0.001), worse physical pain (69 vs. 84%, *p* = 0.037), and a tendency to poorer social relations (61 vs. 75%, *p* = 0.066) in the DHA− group. The Duke scale also showed a lower quality of life in the DHA− group in terms of physical health (58 vs. 72%, *p* = 0.036), perceived health (34 vs. 61%, *p* = 0.043), and general health (47 vs. 62%, *p* = 0.053). There was no significant difference between the two DHA groups in functional impairment evaluated either by the SDS or the GAF.

The evaluation of the psychotic, depressive, or cognitive symptoms based on PANSS, CDSS, and MoCA scores did not show any differences between the two groups (Table 4, Supplementary Table 3). Likewise, the severity of the disease evaluated by the clinician (CGI-S) was in the same range for both groups.

## 4. Discussion

Lipidomic analysis of the RBC membrane of patients from our cohort of 37 individuals showed that 19 (51%) of them displayed a lower level of the ω3 fatty acid DHA compared to the range of the general population. Patients in this DHA− group had higher cortisol and IL-6 levels, and they presented lower quality of life scores and higher number of full-time hospitalizations (Tables 1–3 and Figure 2).

The observed membrane abnormalities in DHA− patients are not likely to result from differences in diet habit or energy metabolism whose markers did not differ between both groups although we lacked data on mitochondrial metabolism. Sex ratio and lifestyle (body mass index, physical activity, and substance use) are not confounding factors either since these parameters were not different between both DHA groups (Supplementary Table 1).

Increased cortisol in the DHA− group suggests a higher level of chronic stress in these patients, or the existence of physio-pathological processes that are costly for organisms with a lower functionality. Increased basal cortisol level is a well-established finding in patients with schizophrenia compared to healthy controls (87–89); in addition, hypercortisolemia was found to trigger psychotic symptoms (90).

IL-6 values were also specifically increased in the DHA− group. In the DHAn group, only one patient presented a weakly positive IL-6 value (1.3 pg/mL) while it was undetectable for the rest of the group. In contrast, IL-6 level was high (>10 pg/mL) for 7 out of the 19 patients in the DHA− group. Strikingly, higher IL-6 level was not associated with any other inflammation markers such as US-CRP, IL-1β, or TNFα. Furthermore, the 7 patients in the DHA− group that displayed higher IL-6 rates had comparable proportion of DHA compared to the whole DHA− group (4.8% in both cases).

The anti-inflammatory properties of DHA have been widely studied. According to some *in vitro* works, DHA would act differentially on TNFα and IL-6 expression, selectively reducing IL-6 expression without any effect on TNFα (91, 92), through the reduction of NFkB activity. This is in line with our results.

The effect of APs on cortisol and IL-6 levels is also a critical issue in particular since APs have been shown to induce IL-6 genes methylation, resulting in lower rates of IL-6 when prescribed in drug-naïve patients suffering from schizophrenia (93). Blood levels of IL-6 and cortisol were also found to be lower upon administration of the AP haloperidol to healthy volunteers (89).

Another important result of our study is the significant differences in quality of life between the two DHA groups. Scores of SF-36 and Duke scales both attested to a poorer physical health, perceived health, physical pain perception, and general health in patients from the DHA− group. There was also a tendency for the DHA− group to perform worse on the social health scale.

In line with the animal study of Abbott et al. (94) we did not find in our cohort any correlation between lifestyle (diet and physical activity) and DHA level in RBC membranes. In agreement with this finding, while 3 patients in our sample received an ω3 supplementation (at around 900 mg/j), 2 of them belonged to the DHA− group. Deficits in ω3 were repeatedly shown to be associated with various cognitive deficits in schizophrenia (95). This finding has been further supported by a recent animal study showing that a diet deficient in ω3 during development was associated with a motivational deficit later in life (96). This would explain the differences observed between the two DHA groups in terms of perceived health.

Finally, the symptomatology did not differ between the two DHA groups, whether at the psychotic, depressive, or cognitive level. There was, however, a small tendency (*p* < 0.2) for more depressive symptoms and less attentional abilities in the DHA− group.

One should note that the increase of AA measured here in patients with schizophrenia is in contradiction with some previous studies that observed a decrease of both AA and DHA in the RBC of patients (30, 31, 51). This may be due to the fact that these studies were conducted on patients in the acute phase and/or in the absence of any AP treatment. In agreement with this, a study in which patients were in a remitted or partly remitted phase of schizophrenia reported higher FA levels in patients compared to controls (54). Our cohort also focused on patients in the stabilization phase, where there might be compensatory or preventive mechanisms, generating a reserve pool of AA that would be quickly consumed during the acute phase. Another explanation may be that AP treatments would correct the decrease of AA, whereas it would not have any effect on DHA levels.

This study has several limitations. First, the small number of patients makes it difficult to draw any definitive conclusions and calls for a multicentric study conducted on a wider sample. Second, we observed that patients in the DHA− group were significantly older, had a significantly lower level of education, and a higher number of hospitalizations. These parameters can be confounding factors, or a sign of more severe illness in the DHA− group. The fact that the DHA− group had a lower level of education could thus be related to the neurodevelopmental hypothesis of schizophrenia. Finally, this study was exploratory, and we acknowledge that such data-driven exploratory study is subject to alpha inflation due to multiple testing.

The present work suggests that specific biological markers such as RBC membrane FA content could be the signature of a specific endophenotype of schizophrenia patients with higher stress and inflammatory response, as well as a lower quality of life. Though preliminary, it could guide non-pharmacological interventions based on biological results, in particular by addressing critical questions such as lifestyle habits and stress management. It could also pave the way for future research directions aiming at using biological markers to optimize the pharmacological treatment of already stabilized patients.

## Data availability statement

The datasets presented in this article are not readily available because datasets are the ownership of Assistance Publique–Hôpitaux de Paris (AP-HP). Requests to access the datasets should be directed to VA, vladimir.adrien@aphp.fr.

## Ethics statement

The studies involving human participants were reviewed and approved by AP-HP Health Data Warehouse (EDS). The patients/participants provided their written informed consent to participate in this study.

## Author contributions

VA designed the protocol of the study under PN supervision. VA, NB, and PN performed data extraction and analysis. VA, HF, DT, and PN wrote the manuscript. CT and SM supervised the development of this work. NB, CT, SM, and FF reviewed the manuscript. All authors contributed to the article and approved the submitted version.

## Footnotes

1
https://www.cdc.gov/epiinfo
