# Supplementary material for: Higher stress response and altered quality of life in schizophrenia patients with low membrane levels of docosahexaenoic acid
Source: Front Psychiatry. 2023 Feb 3;14:1089724. doi: 10.3389/fpsyt.2023.1089724 (PMC9937080; doi:10.3389/fpsyt.2023.1089724)
Supplement: Supplementary file 1 [file Table_1.pdf]

Table S1. Results on lifestyle (diet habits and physical activity) for the DHAn and DHA- groups. Standard deviations are between brackets. There are no statistical differences between the two groups.

	**Total**	**DHAn (n=18)**	**DHA- (n=19)**	**p-value**
Diet				
Bread	2.8 (2.0)	3.0 (2.3)	2.5 (1.8)	0.49
Feculents	3.4 (2.3)	3.7 (2.2)	3.2 (2.5)	0.47
Dried vegetables	3.3 (2.2)	3.2 (2.3)	3.3 (2.2)	0.90
Milk products	2.8 (1.5)	3.0 (1.7)	2.6 (1.3)	0.46
Fruits	3.1 (2.1)	3.1 (2.2)	3.2 (2.2)	0.89
Vegetables	3.5 (2.4)	3.3 (2.4)	3.0 (2.5)	0.68
Meat	3.5 (2.4)	3.6 (2.6)	3.4 (2.1)	0.76
Fish	2.6 (2.0)	2.9 (2.0)	2.3 (1.9)	0.34
Ready-made dishes	5.9 (2.2)	5.9 (2.3)	5.8 (2.1)	0.94
Sweet products	4.0 (2.4)	4.5 (2.4)	3.5 (2.4)	0.23
Physical activity				
Total activity	20.5 (6.0)	20.2 (5.4)	20.8 (6.7)	0.76
Leisure activity	10.3 (5.0)	10.4 (4.7)	10.3 (5.5)	0.94
Sport	7.8 (2.5)	7.5 (2.4)	8.2 (2.7)	0.44
Sedentarity	2.4 (1.4)	2.3 (1.3)	2.4 (1.5)	0.85
Hours of TV/day	2.6 (2.9)	3.2 (3.8)	2.1 (1.5)	0.22
