# Supplementary material for: Higher stress response and altered quality of life in schizophrenia patients with low membrane levels of docosahexaenoic acid
Source: Front Psychiatry. 2023 Feb 3;14:1089724. doi: 10.3389/fpsyt.2023.1089724 (PMC9937080; doi:10.3389/fpsyt.2023.1089724)
Supplement: Supplementary file 2 [file Table_2.pdf]

Table S2. Results on metabolic-energetic markers for the DHAn and DHA- groups. BA: bile acids. AA: amino-acids. Standard deviations are between brackets. There are no statistical differences between the two groups.

	**Total**	**DHAn (n=18)**	**DHA- (n=19)**	**p-value**
Triglycerids	1.64 (0.91)	1.75 (1.06)	1.54 (0.76)	0.49
HDL-cholesterol	0.47 (0.16)	0.47 (0.20)	0.47 (0.12)	0.96
LDL-cholesterol	1.11 (0.28)	1.11 (0.29)	1.12 (0.28)	0.92
Primary BA	0.72 (2.03)	0.43 (0.62)	0.99 (2.77)	0.41
Secondary BA	0.50 (0.84)	0.35 (0.24)	0.64 (1.14)	0.31
Total BA	1.24 (2.99)	0.78 (0.72)	1.68 (4.13)	0.37
Non conjugated BA	0.44 (0.59)	0.38 (0.49)	0.50 (0.69)	0.52
Vitamine B6	47.9 (42.0)	52.5 (40.1)	43.5 (44.4)	0.52
Vitamine B9	16.1 (7.3)	14.6 (8.0)	17.5 (6.4)	0.22
Vitamine B12	295.5 (78.3)	310.1 (94.2)	281.6 (58.8)	0.28
Methionine	23.5 (4.1)	23.8 (3.7)	23.3 (4.5)	0.74
Homocysteine	14.7 (6.2)	14.7 (7.3)	14.7 (5.3)	0.97
Tryptophan	47.9 (10.9)	48.7 (12.1)	47.1 (9.8)	0.66
Total AA	3140 (376)	3100 (318)	3178 (429)	0.53
