# Supplementary material for: Higher stress response and altered quality of life in schizophrenia patients with low membrane levels of docosahexaenoic acid
Source: Front Psychiatry. 2023 Feb 3;14:1089724. doi: 10.3389/fpsyt.2023.1089724 (PMC9937080; doi:10.3389/fpsyt.2023.1089724)
Supplement: Supplementary file 3 [file Table_3.pdf]

Table S3. Results on the PANSS symptoms for the DHAn and DHA- groups. P is for positive symptoms, N for negative symptoms and G for General psychopathology symptoms. Standard deviations are between brackets. There are no statistical differences between the two groups.

	Total	DHAn (n=18)	DHA- (n=19)	p-value
P1	3.4 (1.7)	3.4 (1.8)	3.4 (1.7)	0.96
P2	2.7 (0.7)	2.6 (0.6)	2.7 (0.8)	0.60
P3	2.5 (1.7)	2.3 (1.7)	2.6 (1.7)	0.60
P4	2.1 (1.1)	1.9 (1.2)	2.2 (1.0)	0.56
P5	1.8 (1.2)	2.1 (1.4)	1.5 (1.0)	0.19
P6	3.1 (1.7)	3.3 (1.4)	2.8 (2.0)	0.44
P7	2.5 (1.1)	2.9 (1.0)	2.2 (1.2)	0.071
N1	3.9 (1.3)	4.2 (1.2)	3.6 (1.4)	0.18
N2	3.7 (1.1)	3.7 (1.1)	3.7 (1.2)	0.97
N3	2.8 (0.9)	2.8 (1.0)	2.7 (0.8)	0.63
N4	3.1 (0.9)	3.0 (0.8)	3.2 (1.1)	0.61
N5	3.2 (1.7)	3.0 (1.5)	3.3 (1.9)	0.57
N6	2.6 (1.3)	2.9 (1.5)	2.4 (1.1)	0.29
N7	2.4 (0.9)	2.3 (0.9)	2.5 (1.0)	0.65
G1	2.3 (1.5)	2.2 (1.5)	2.4 (1.6)	0.70
G2	2.7 (1.1)	2.6 (1.1)	2.8 (1.0)	0.51
G3	3.2 (1.3)	3.4 (1.0)	3.1 (1.4)	0.35
G4	2.5 (1.0)	2.6 (0.8)	2.3 (1.1)	0.37
G5	2.9 (0.8)	3.0 (0.5)	2.7 (0.9)	0.29
G6	3.0 (1.5)	3.1 (1.4)	2.9 (1.6)	0.83
G7	2.4 (1.0)	2.6 (1.0)	2.3 (1.0)	0.40
G8	2.1 (1.0)	2.1 (1.1)	2.1 (0.9)	0.88
G9	3.0 (1.3)	2.9 (1.3)	3.1 (1.4)	0.81
G10	1.8 (1.1)	1.7 (1.1)	1.8 (1.1)	0.85
G11	2.9 (1.0)	3.1 (0.9)	2.6 (1.1)	0.16
G12	2.4 (1.3)	2.5 (1.2)	2.2 (1.3)	0.49
G13	2.8 (1.2)	2.7 (1.2)	2.9 (1.1)	0.66
G14	2.2 (1.0)	2.1 (1.1)	2.2 (0.9)	0.76
G15	2.1 (0.9)	2.2 (0.9)	2.1 (0.9)	0.84
G16	3.2 (1.4)	3.1 (1.2)	3.4 (1.6)	0.44
